# Sex-specific efficacy and safety outcomes in patients with resectable stage III non-small-cell lung cancer (NSCLC) undergoing neoadjuvant therapies: a pooled analysis of the SAKK trials 16/96, 16/00, 16/01, 16/08 and 16/14

**DOI:** 10.1016/j.esmoop.2025.105870

**Published:** 2025-11-10

**Authors:** L. Frehner, S. Schär, S. Hayoz, M. Guckenberger, T. Finazzi, M. Mark, A. Addeo, L.A. Mauti, D. Betticher, R. Stupp, A. Curioni-Fontecedro, S. Peters, M. Früh, S.I. Rothschild, M. Pless, D. König, I. Opitz, H.B. Ris, B.C. Özdemir, S. Schmid

**Affiliations:** 1 Department of Medical Oncology, Inselspital, Bern University Hospital, University of Bern, Bern, Switzerland; 2 Swiss Group for Clinical Cancer Research (SAKK) Competence Center, Bern, Switzerland; 3 Department of Radiation Oncology, University Hospital Zurich, University of Zurich, Zurich, Switzerland; 4 Department of Radiation Oncology, Cantonal Hospital Baden, Baden, Switzerland; 5 Division of Oncology/Hematology, Cantonal Hospital Graubuenden, Chur, Switzerland; 6 Università della Svizzera italiana, Faculty of Biomedical Sciences, Lugano, Switzerland; 7 Department of Oncology, University Hospital Geneva, Geneva, Switzerland; 8 Department of Medical Oncology, Kantonsspital Winterthur, Winterthur, Switzerland; 9 Clinic of Oncology, Cantonal Hospital Fribourg, Fribourg, Switzerland; 10 Faculty of Science and Medicine, University of Fribourg, Fribourg, Switzerland; 11 Lurie Comprehensive Cancer Center, Northwestern University Feinberg School of Medicine, Chicago, USA; 12 Department of Medical Oncology, Centre Hospitalier Universitaire Vaudois (CHUV) and University of Lausanne, Lausanne, Switzerland; 13 Department of Oncology, Centre Hospitalier Universitaire Vaudois (CHUV), Lausanne, Switzerland; 14 Department of Medical Oncology and Hematology, HOCH Health Ostschweiz, Kantonsspital, St. Gallen, Switzerland; 15 Center of Oncology/Hematology and Comprehensive Cancer Center, Cantonal Hospital Baden, Baden, Switzerland; 16 Department of Medical Oncology, University Hospital Basel, Basel, Switzerland; 17 Department of Thoracic Surgery, University Hospital Zurich, University of Zurich, Zurich, Switzerland; 18 Clinics for Thoracic Surgery, Hôpital du Valais, Sion, Switzerland

**Keywords:** non-small-cell lung cancer, stage III, sex-specific survival, surgical outcomes

## Abstract

**Background:**

Current data suggest better survival in various cancer types but increased treatment toxicity in female compared with male patients. In this article, we report a pooled analysis of sex-related differences in survival outcomes and safety in patients with resectable stage III non-small-cell lung cancer (NSCLC) treated in five prospective clinical trials.

**Patients and methods:**

Data from 499 patients included in five Swiss Group for Clinical Cancer Research (SAKK) trials for resectable stage III NSCLC were pooled. All patients were treated with three cycles of chemotherapy (cisplatin/docetaxel), either alone (*n* = 207, 41%), with sequential radiotherapy (*n* = 229, 46%) or with sequential perioperative programmed death-ligand 1 blockade (*n* = 62, 12%).

**Results:**

Of 499 patients included, 341 (68.3%) were male. Median event-free survival (EFS) [24.4 versus 11.8 months, hazard ratio (HR) 1.32, 95% confidence interval (CI) 1.06-1.64, *P* = 0.014] and median overall survival (OS) (59.3 versus 26.1 months, HR 1.45, 95% CI 1.15-1.83, *P* = 0.0018) were significantly longer in female patients compared with male patients. OS/EFS remained significant in a multivariable Cox regression model. While the cause-specific hazard of non-cancer-related death was increased in males (HR 2.14, 95% CI 1.32-3.46, *P* = 0.0019), the risk of tumor-related death was not significantly different between sexes (HR 1.26, 95% CI 0.96-1.65, *P* = 0.09). No significant differences in treatment-related grade ≥3 adverse events (62.5% versus 69.7%) or treatment discontinuation (3.2% versus 3.2%) were observed.

**Conclusion:**

In this pooled analysis, female patients with resectable stage III NSCLC had longer EFS and OS than males, mainly due to lower non-cancer-related mortality. Given the retrospective design and limited sample size, these results should be interpreted with caution. Prospective studies are needed to confirm these findings and explore underlying causes of sex-based differences.

## Highlights

• Females with resectable stage III NSCLC treated with neoadjuvant therapies have longer OS and EFS.
• Severe treatment-related toxicities do not differ significantly between sexes.
• Male patients have a higher risk of non-cancer-related deaths than females.

## Introduction

The cornerstone of treatment of resectable non-small-cell lung cancer (NSCLC) is surgery. For patients with stage II and III NSCLC, additional systemic treatment is recommended in either the neoadjuvant or adjuvant setting with a significant though modest overall survival (OS) benefit associated with platinum-based chemotherapy.^1^^,^^2^ Recently, several phase III trials have investigated immune checkpoint inhibitor (ICI)-based treatment combinations in the neoadjuvant, adjuvant or perioperative setting.^3^, ^4^, ^5^, ^6^, ^7^, ^8^, ^9^ Overall, the addition of a checkpoint inhibitor to platinum-based chemotherapy was associated with a significant benefit in event-free survival (EFS) and—in those trials with a neoadjuvant component—rate of pathological complete responses (pCR). Furthermore, a significant OS benefit was observed for perioperative pembrolizumab added to neoadjuvant platinum-based chemotherapy in the KEYNOTE 671 trial.^3^ A recent press release also reported a significant OS benefit for neoadjuvant nivolumab in combination with chemotherapy in the CheckMate (CM) 816 trial.^10^ These findings establish neoadjuvant chemo-immunotherapy with or without additional adjuvant ICI treatment as a new standard of care for resectable NSCLC.

There is an increasing awareness on the potential impact of both, biological sex and sociocultural gender on cancer risk and outcomes. To address this, the European Society for Medical Oncology (ESMO) has established the ESMO Gender Medicine Task Force, aiming to educate and raise awareness within the oncology community about incorporating sex and gender considerations into trial design and analysis.^11^ Across different cancer types and populations, women generally have a lower cancer risk and better survival rates for various cancer types compared with men.^12^ Globally, female patients are at greater risk of experiencing toxicity from various drug classes,^13^ including anticancer therapies, with an increased rate of both severe hematological and non-hematological adverse events (AEs), such as mucositis and nausea.^14^

In advanced NSCLC, patient sex is an important prognostic factor. A sex-specific analysis of efficacy and toxicity of over 1100 patients treated with one of four different platinum doublet regimens in the Eastern Cooperative Oncology Group (ECOG) 1594 trial revealed that despite similar response rates and greater toxicity, female patients had significantly improved OS compared with male patients.^15^ A larger pooled analysis of five randomized phase III chemotherapy trials for stage IIIA-IV NSCLC confirmed that females with adenocarcinoma histology had an OS advantage after adjusting for age, stage and performance stage.^16^ A retrospective analysis of three phase III NSCLC trials conducted by the National Cancer Institute of Canada Clinical Trials Group was carried out to assess the effect of sex on efficacy, AEs, dose intensity and quality of life (QoL) in a heterogeneous group of patients with stage IB-IV disease treated with different platinum-containing regimens. Progression-free survival was found to be moderately prolonged in female patients with chemotherapy-treated NSCLC; however, no significant difference in OS or QoL was observed and the rates of serious AEs (SAEs) were comparable.^17^

In contrast to chemotherapy, conflicting results exist on sex differences in survival benefit from ICIs for stage IV NSCLC. Besides retrospective single-center analyses which showed similar efficacy but higher risk of immune-mediated AEs with ICI treatment for female patients,^18^^,^^19^ a systematic review and meta-analysis revealed a greater OS benefit in the female population with the addition of ICIs to chemotherapy compared with male patients [pooled hazard ratio (HR) for OS 0.69 versus 0.8].^20^ In contrast, a similar meta-analysis indicated a significantly greater effect on OS for male patients than for females with first-line ICI monotherapy compared with chemotherapy for NSCLC with high programmed death-ligand 1 (PD-L1) expression.^21^ However, in a meta-analysis investigating the association between patient sex with ICI efficacy and OS in advanced cancers, including 11 NSCLC trials, no sex differences in OS were found,^22^ highlighting the need for further investigation.

In the curative setting data on sex-related differences in survival outcomes and safety are scarce. A recent meta-analysis showed significantly higher major pathological response (MPR) rates and a trend for higher pCR rates in female NSCLC patients^23^; however, the impact of sex on survival and toxicity is unknown.

The Swiss Group for Clinical Research (SAKK) carried out several prospective trials in patients with resectable stage III NSCLC. These trials (SAKK 16/96, SAKK 16/00, SAKK 16/01, SAKK 16/08 and SAKK 16/14)^24^, ^25^, ^26^, ^27^, ^28^ investigated neoadjuvant platinum-based chemotherapy, either alone or combined with other agents and/or radiotherapy. All reported efficacy and safety results for the overall population. However, sex-specific analyses for efficacy (e.g. EFS, pathological response and OS) and safety outcomes (e.g. rate and type of AEs, treatment discontinuation rate) were not carried out.

The overall aim of this pooled analysis was to evaluate potential sex differences in surgical and survival outcomes as well as safety in patients with resectable NSCLC receiving neoadjuvant systemic therapy.

## Patients and methods

### Study design and treatment

Patients with stage III NSCLC enrolled in the five SAKK trials 16/96, 16/00, 16/01, 16/08 and 16/14 were included in this analysis. The detailed study designs, inclusion and exclusion criteria and methods of these trials have been published previously.^24^, ^25^, ^26^, ^27^, ^28^

In brief, all studies included patients with resectable stage IIIA N2 and IIIB NSCLC that could be encompassed in a single radiation port. The trials investigated different neoadjuvant treatment regimens on a backbone of three cycles of cisplatin/docetaxel chemotherapy (cisplatin 100 mg/m^2^ and docetaxel 85 mg/m^2^, given once every 3 weeks) followed by surgery. In the trials SAKK 16/00 (arm A) and 16/01, patients underwent neoadjuvant short-course preoperative accelerated radiotherapy over 3 weeks (44 Gy in 22 fractions) after completion of chemotherapy. Additional concomitant cetuximab during both chemo- and radiotherapy was given in SAKK 16/08 (loading dose 400 mg/m^2^, followed by 250 mg/m^2^). In SAKK 16/14 patients received one dose of neoadjuvant durvalumab (750 mg/dose, biweekly) after completion of chemotherapy (no radiotherapy) and 1 year of adjuvant durvalumab. Surgery included an anatomical tumor resection with mediastinal lymph node dissection.^29^

The trials were conducted within the Swiss multicentric SAKK network. SAKK 16/00 additionally included participants from two European partner institutions. All studies were carried out in accordance with the principles of the Declaration of Helsinki and the guidelines on Good Clinical Practice. The protocols were approved by local ethics committees. Written informed consent was obtained from all patients.

### Patient population

Overall, 505 patients were enrolled in five consecutive SAKK trials: 90 patients in 16/96, 232 in 16/00, 46 in 16/01, 69 in 16/08 and 68 in the SAKK 16/14. The first patient in SAKK 16/96 was included in April 1997, and the last patient in SAKK 16/14 was included in January 2019. For the present analysis, six patients were excluded: two patients were retrospectively detected to have stage IV disease at diagnosis and the others had stage IIB disease with the adapted seventh tumor–node–metastasis (TNM) staging edition.

Details of the staging procedures were described in the respective studies. Pathological staging was carried out by mediastinoscopy or endobronchial ultrasonography, if available. Since the introduction of [^18^F]2-fluoro-2-deoxy-d-glucose (FDG) positron emission tomography (PET) into clinical routine in the early 2000s, 326 (65%) patients have had a baseline FDG–PET/computed tomography scan. SAKK 16/96, 16/00 and 16/14 enrolled patients with T1-3 N2 stage, whereas SAKK 16/01 and 16/08 enrolled patients with T4 N0-3 or T1-4 N3 stage. The fifth (16/96) and sixth (16/00, 16/01 and 16/08) versions of the TNM staging system in use at the time have been translated into the seventh version for the purposes of this analysis.

### Outcomes

Response and follow-up assessments for each of the five SAKK studies were carried out according to the specific study protocol. All patients were followed up for OS and EFS. Data cut-off for the present study was 17 January 2024.

Efficacy and safety outcomes were assessed in female patients (cohort A) and male patients (cohort B) for comparison purposes. Efficacy outcomes included median EFS, median OS and OS at defined time points (3 years, 5 years and 10 years), and pCR. EFS was defined as the time from registration or randomization to objective tumor progression or relapse, secondary tumor (16/00 and 16/14) or death due to any cause, whichever occurred first. OS was the interval from the date of registration or randomization until the date of death from any cause. Patients who did not experience an event were censored at the date last known alive. Rates of deaths due to tumor progression and deaths due to other reasons were assessed and compared between sexes.

pPCR was defined as ≥95% necrosis or fibrosis in the trial SAKK 16/96 and absence of vital tumor cells (0%) in the four other trials. Safety outcomes included assessment of SAEs and treatment modification due to toxicity (all trials) and treatment-related grade ≥3 AEs (SAKK 16/08 and SAKK 16/14). We also looked at sex-specific surgical outcomes, including the rate of complete resection and perioperative mortality.

### Statistical analysis

Continuous variables were summarized by median and range. Categorical variables were summarized using frequency counts and percentages. Standard tests were used to check univariable associations between categorical and categorical (Fisher’s exact tests or chi-square tests) or categorical and continuous (Wilcoxon rank sum test) variables in the frame of subgroup analysis. Logistic regression was used to test multivariable associations between binary outcomes and other patient characteristics. For time-to-event endpoints, Kaplan–Meier estimates and corresponding 95% confidence intervals (CIs) based on the log–log approach were used to describe and visualize the effect of categorical variables. Between-group survival curves and rates were compared using the log-rank test and the Kaplan–Meier method at a specific time point, respectively. We analyzed potential prognostic factors for EFS and OS using univariable and multivariable Cox regression models. This analysis included sex (female versus male), age (continuous variable), smoking status (never smoker versus current/former [all tobacco]), histology (non-squamous versus squamous) and treatment modality (neoadjuvant chemotherapy versus neoadjuvant chemoradiotherapy versus neoadjuvant chemo-immunotherapy). The proportional hazards assumption was checked by testing the scaled Schoenfeld residuals. Interactions between covariables were assessed using likelihood ratio tests. Competing risk analysis for OS (death due to tumor versus other cause) was carried out using cause-specific hazard models.

The threshold for statistical significance was *P* < 0.05. All analyses were carried out in SAS v. 9.4 and R version 4.2.3.

## Results

### Study population and treatment

Among 499 patients included in this pooled analysis, 158 (31.7%) were female and 341 (68.3%) were male. Patient and disease characteristics are summarized in Table 1. Median age was 60 years (range 28-76 years), and most patients were current or former smokers (93%) and had an ECOG performance status (PS) of 0-1 (97%). Tumors were classified as squamous cell (36%) and non-squamous cell (64%). Overall, 84% of patients had N2 and 85% had stage IIIA disease, whereas 15% had stage IIIB.

**Table 1:** Baseline characteristics

Characteristic	Overall, *N* = 499	F, *N* = 158	M, *N* = 341	*P* value^a^
Trial, *n* (%)				0.015
SAKK 16/96	88 (18)	21 (13)	67 (20)	
SAKK 16/00	231 (46)	76 (48)	155 (45)	
SAKK 16/01	43 (8)	13 (8)	30 (9)	
SAKK 16/08	69 (14)	16 (10)	53 (16)	
SAKK 16/14	68 (14)	32 (20)	36 (11)	
Age at registration, years (range)	60 (28-76)	59 (28-76)	60 (30-76)	0.4
Smoking status, *n* (%)				<0.001
Current/former	463 (93)	136 (86)	327 (96)	
Never	36 (7)	22 (14)	14 (4)	
Histology, *n* (%)				<0.001
Non-squamous	319 (64)	124 (78)	195 (57)	
Squamous	180 (36)	34 (22)	146 (43)	
ECOG PS, *n* (%)				0.7
0	330 (66)	107 (68)	223 (65)	
1	157 (31)	47 (30)	110 (32)	
2	1 (0.2)	0 (0)	1 (0.3)	
(Missing)	11 (2)	4 (2)	7 (2)	
Nodal status (TNM seventh edition), *n* (%)				0.4
N0	32 (6)	6 (4)	26 (8)	
N1	5 (1)	2 (1)	3 (1)	
N2	417 (84)	137 (87)	280 (82)	
N3	45 (9)	13 (8)	32 (9)	
T-stage (TNM seventh edition), *n* (%)				0.2
T1	66 (13)	24 (15)	42 (12)	
T2	225 (45)	78 (49)	147 (43)	
T3	130 (26)	37 (23)	93 (27)	
T4	78 (16)	19 (12)	59 (17)	
UICC stage (TNM seventh edition), *n* (%)				0.4
IIIA	422 (85)	137 (87)	285 (84)	
IIIB	77 (15)	21 (13)	56 (16)	
Neoadjuvant treatment, *n* (%)				0.008
Chemotherapy only	207 (41)	59 (37)	148 (43)	
Chemo-immunotherapy	62 (12)	31 (20)	31 (9)	
Chemoradiotherapy	229 (46)	68 (43)	161 (47)	
None	1 (0.2)	0 (0)	1 (0.3)	

ECOG, Eastern Cooperative Oncology Group; F, female; M, male; PS, performance status; SAKK, Swiss Group for Clinical Cancer Research; TNM, tumor–node–metastasis.

a Pearson’s Chi-squared test; Wilcoxon rank sum test; Fisher’s exact test.

Female patients were more likely to be never smokers (14% versus 4%, *P* ≤ 0.001) and have non-squamous histology (78% versus 57%, *P* ≤ 0.001). Other baseline characteristics such as ECOG PS (ECOG PS 0: 68% versus 65%, *P* = 0.7) and nodal stage (N0 3.8% versus 7.6%, N1 1.3% versus 0.9%, N2 87% versus 82%, N3 8.2% versus 9.4%, *P* = 0.4) were balanced between sexes as seen in Table 1. Overall, 207 patients (41%) were assigned to neoadjuvant chemotherapy only, 62 patients (12%) to neoadjuvant sequential chemo-immunotherapy and 229 patients (46%) to neoadjuvant sequential chemoradiotherapy. The relatively high proportion of females in the SAKK 16/14 trial (47% females) translated into a significant difference in the treatment distribution by sex, with a higher proportion of females receiving neoadjuvant chemo-immunotherapy compared with males in the overall pooled population (20% versus 9%, *P* = 0.008).

### Efficacy

At the time of data cut-off (17 January 2024) the median duration of follow-up was 7.8 years. Overall, 47 (30%) of the 158 female patients and 73 (21%) of the 341 male patients were alive and remained free from recurrence.

**Figure 1:**
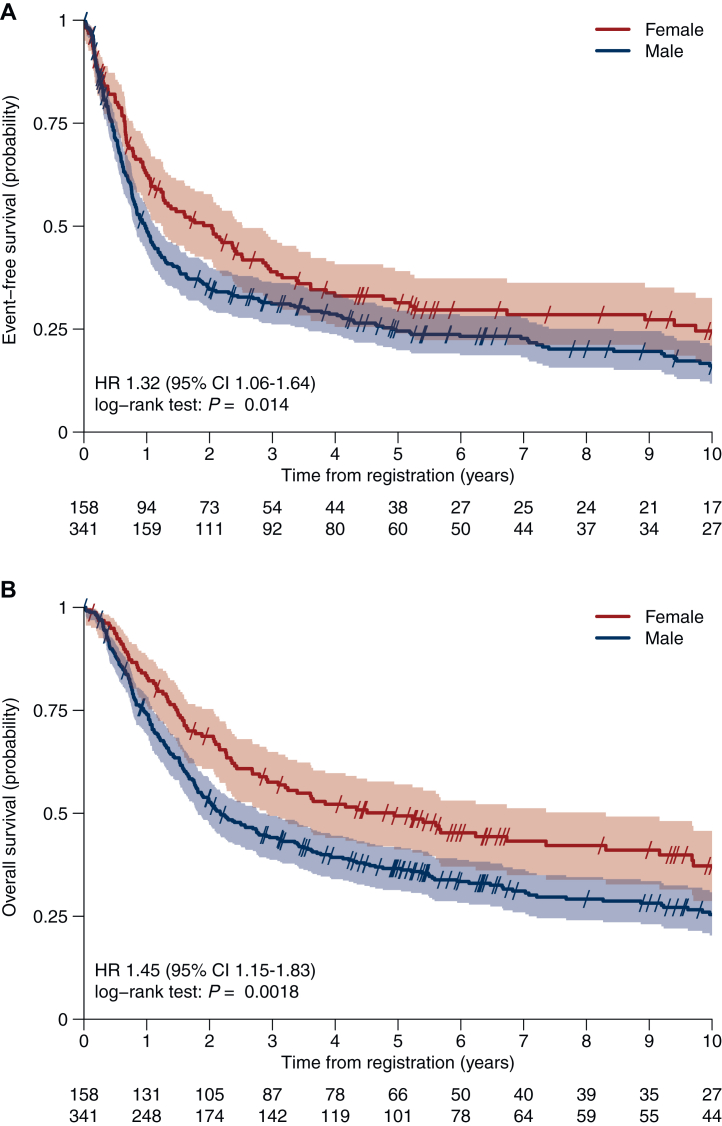
**Sex-specific event-free survival and overall survival.** Sex-specific event-free survival (years) (A) and overall survival (years) (B) in the overall pooled population. CI, confidence interval; HR, hazard ratio.

The median EFS was significantly longer in female patients compared with male patients with 24.4 versus 11.8 months (HR 1.32, 95% CI 1.06-1.64, *P* = 0.014) (Figure 1). The difference between sexes in EFS remained significant in a multivariable Cox regression model correcting for age, treatment modality, smoking status and histology (HR 1.35, 95% CI 1.07-1.7, *P* = 0.011) as shown in Supplementary Figure S1, available at https://doi.org/10.1016/j.esmoop.2025.105870.

Median OS was also significantly longer in females (59.3 versus 26.1 months, HR 1.45, 95% CI 1.15-1.83, *P* = 0.0018) (Figure 1) with 3-, 5- and 10-year OS rates of 58% versus 44%, 49% versus 37% and 37% versus 25%, respectively. This benefit remained significant after correcting for age, smoking status, treatment modality and histology (HR 1.38, 95% CI 1.08-1.76) (Supplementary Figure S2, available at https://doi.org/10.1016/j.esmoop.2025.105870). The sex-specific difference in EFS and OS remains significant also when corrected for trials instead of treatment modality in the multivariable analysis (Supplementary Table S1, available at https://doi.org/10.1016/j.esmoop.2025.105870).

The primary cause of death in the study cohort was tumor progression, accounting for death events in 251 patients (72%), including 78 females (80%) and 173 males (69%). Overall, 74 (21%) patients died of causes other than tumor progression, with a higher rate seen in males (23%) compared with females (16%).

**Figure 2:**
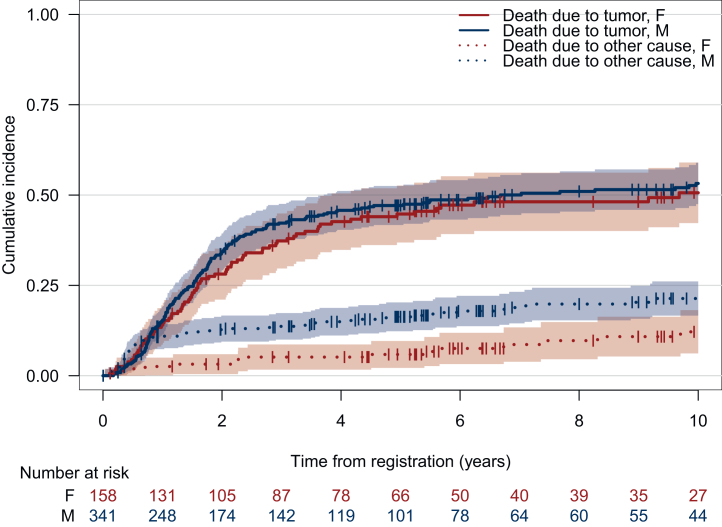
**Cumulative risk incidence for death according to sex.** Cumulative incidence for death due to tumor and death due to other cause for female and male patients. F, females; M, males.

Risk of death due to tumor progression did not significantly differ between males and females (HR 1.26, 95% CI 0.96-1.65, *P* = 0.09), indicating no substantial sex-related difference in tumor-related mortality. However, the risk of death from causes other than tumor progression was significantly higher in males compared with females, with a HR of 2.14 (95% CI 1.32-3.46, *P* = 0.0019) as illustrated in cumulative incidence functions for the risk of tumor-related death and death due to other causes in Figure 2.

Among deaths due to causes other than tumor progression, the most common were cardioembolic events, accounting for 22% (16% in females versus 24% in males), followed by secondary cancers (11%; 21% in females versus 9% in males). Other notable causes included infections (8%) and respiratory issues (5%). Data on the specific cause of death were missing for 32% of the patients (Supplementary Table S2, available at https://doi.org/10.1016/j.esmoop.2025.105870).

### Patterns of relapse and subsequent treatment

A total of 314 patients (63%) experienced disease recurrence, 284 (90%) presenting with distant recurrence and 27 (9%) with local recurrence only. For three patients (1%), the pattern of recurrence could not be determined anymore. Local recurrence only was relatively more frequent in males compared with females (12% versus 2%, *P* = 0.004) (Supplementary Table S3, available at https://doi.org/10.1016/j.esmoop.2025.105870).

Post-relapse treatment data were prospectively evaluated in the SAKK 16/08 (69 patients) and SAKK 16/14 (68 patients) trials, and partially in the SAKK 16/00 trial (231 patients). Of the 226 (61%) patients with recurrence in these trials, 120 patients (53%) received at least one line of systemic therapy while 45 patients (20%) received local radiotherapy, with no significant differences between females (54% and 24%) and males (53% and 18%) (*P* = 0.8 for further systemic therapy and *P* = 0.2 for further radiotherapy) (Supplementary Table S4, available at https://doi.org/10.1016/j.esmoop.2025.105870).

### Safety

The incidence of treatment-related SAEs was significantly higher in males (13.5% versus 6.3%, *P* = 0.021). SAKK 16/08 and SAKK 16/14 also assessed AEs according to Common Terminology Criteria for Adverse Events 3.0 and 4.03, respectively. In these two trials, the incidence of treatment-related AEs of grade ≥3 was comparable between sexes (*P* = 0.448).

Dose modifications of the systemic therapy due to toxicity in the overall study population were significantly more frequent in female than in male patients (32% versus 23%, *P* = 0.037). However, the rate of treatment discontinuation due to toxicity was similar in both cohorts and occurred in 3% of both, females and males (5 and 11 patients, respectively) (Table 2).

**Table 2:** Incidence of treatment-related grade 3 AE in SAKK 16/08 und 16/14 and dose modification and treatment discontinuation due to toxicity in the overall population

Variable	F, *N* = 48	M, *N* = 89	*P* value^a^
Treatment-related AE (G ≥ 3) in SAKK 16/08 and 16/14	30 (62.5%)	62 (70%)	0.448

Variable	F, *N* = 158	M, *N* = 341	*P* value
Treatment failure	5 (3%)	11 (3%)	>0.9
Dose modification	51 (32%)	80 (23%)	0.037

AE, adverse event; F, female; M, male; SAKK, Swiss Group for Clinical Cancer Research.

a Fisher’s exact test; Pearson’s Chi-squared test.

### Surgical outcomes

One hundred and twenty-seven (80%) of the female patients and 270 (79%) of the male patients underwent surgery. A complete resection (R0) was obtained in 109 (69%) of the female and 214 (63%) of the male patients, respectively. Twenty-seven (17.1%) female and 49 (14.4%) male patients achieved a pCR (*P* = 0.4). The R1 rate, indicating microscopically positive surgical margins, was similar in both groups (females 8% versus males 7%, *P* = 0.92). Pneumonectomy was carried out in 20 females (13%) and 77 males (23%) (*P* = 0.0009). The median duration of hospital stay after surgery was 12 days in both groups (females 6-76 days versus males 3-134 days), with no significant difference between sexes (*P* = 0.96). The 30-day mortality (one female, three males) as well as the 60-day mortality (two females, four males) was 1% in both groups (*P* = 0.77 and *P* = 0.93, respectively) (Supplementary Table S5, available at https://doi.org/10.1016/j.esmoop.2025.105870). The attrition rate was similar between sexes with 31 (20%) of the female patients and 71 (21%) of the male patients not undergoing surgery.

## Discussion

Despite the increasing interest on the impact of sex and gender on anticancer treatment efficacy, few studies assessed sex-specific outcomes of resectable NSCLC patients undergoing neoadjuvant therapies. Our pooled analysis of individual patient data from five multicenter clinical trials evaluating different neoadjuvant concepts in patients with stage III NSCLC indicates a significantly longer EFS and OS for female patients compared with male patients. These results confirm previous reports showing a female OS and EFS advantage in NSCLC patients of different stages undergoing resection.^30^^,^^31^ Of note, our cohort consisted exclusively of stage III patients while most published data reported on patients with stage I-III.

In our cohort, the median EFS was 24.4 months in females and therefore significantly longer than in males with only 11.8 months. Furthermore, median OS was also significantly longer in female patients than in males translating into increased long-term survival rates. However, cancer-specific survival was similar in both sexes.

Historically, survival differences between sexes were mainly attributed to the greater incidence of adenocarcinomas among female patients and consequently potential differences in tumor biology^30^ given that the effect of age and smoking status was not consistent. Also, according to an analysis of stage I-II NSCLC patients diagnosed between 1991 and 1999 from the SEER registry, female patients had improved lung cancer-specific survival as well as OS independent of treatment type and after adjusting for smoking status, suggesting that NSCLC in female patients has a different natural course.^32^

In our analysis we found that female sex remained significantly associated with improved EFS and OS even after adjusting for histology as well as additional important determinants of relapse and survival such as age, smoking status and treatment modality. Reasons for this remain to be elucidated. Females generally exhibit stronger immune responses than males, partly due to hormonal effects on T-cell activity and programmed cell death protein 1/PD-L1 pathways, while males often have higher tumor mutational burden.^33^ These differences may influence ICI efficacy, with some evidence suggesting greater benefit from chemo–ICI combinations in females and from ICI monotherapy in males.^33^ Since only a minority of our cohort received perioperative PD-L1 blockade, the survival differences observed are unlikely to be solely driven by ICIs, underscoring the need for prospective, biomarker-based studies to better understand these mechanisms.

Interestingly, the increased EFS among female patients in our pooled analysis did not translate into longer lung cancer-specific survival. Despite a doubling of the EFS (24.4 versus 11.8 months, HR 1.32, 95% CI 1.06-1.64, *P* = 0.014), the mortality due to tumor progression was not significantly lower among female patients indicating no substantial sex-related difference in tumor-related mortality. Differences in the rate of systemic therapies received upon relapse were ruled out and therefore do not explain why the later relapse in female patients does not result in improved lung cancer survival. However, the type and duration of systemic therapy during the follow-up period was not fully documented, with a lack of data on molecular alterations and the use of targeted therapy. Interestingly, we observed a significant difference in relapse patterns, with more male patients presenting with locoregional relapse potentially amenable to renewed curative intent treatment compared with females.

We found a prominent increase in non-cancer-related mortality among male patients with cardioembolic events representing the most common cause of non-cancer-related death (24% in males and 16% in females). Therefore, in our analysis non-cancer-related mortality as opposed to lung cancer-specific mortality seems to be the driving force of overall differences in survival between sexes, possibly due to underlying comorbidities likely due to higher tobacco consumption. Unfortunately, data on comorbidities, including more detailed information on smoking habits, were not assessed systematically in all trials, prohibiting further investigation of this hypothesis. However, in agreement with other studies,^34^ smoking status was not associated with survival overall. Notably, among deaths from causes other than tumor progression, a substantial proportion were due to secondary cancers, with a higher incidence in female patients (21% versus 9%). These findings underscore the importance of a long-term follow-up, including preventive strategies and screening for secondary malignancies and other treatment-related comorbidities in cancer survivors.^35^

Rate of pCR has served as co-primary or secondary endpoint in several current randomized phase III trials investigating neoadjuvant chemo-immunotherapy regimens.^3^, ^4^, ^5^, ^6^, ^7^ The pCR rate as well as rate of MPR have emerged as relevant prognostic factors. However, only some of the trials (CM816, CM77T and Aegean) reported pCR rates according to sex with no relevant differences observed. In line with this, pCR and MPR rates in our analysis did not differ significantly between sexes. However, in a retrospective review of the National Cancer Database evaluating survival outcomes in patients with stage I-III NSCLC who achieved a pCR after neoadjuvant chemotherapy with or without radiation between 2004 and 2014, female sex as well as younger age and number of resected lymph nodes were associated with improved OS in multivariable Cox regression modelling,^34^ suggesting improved outcomes in female patients as compared with male patients in the favorable subgroup of patients actually achieving a pCR.

Sex-segregated data on surgical outcomes after neoadjuvant therapies are largely lacking with none of the above-mentioned phase III neoadjuvant or perioperative chemo-immunotherapy trials reporting surgical outcomes according to sex. In a large Chinese cohort (with only 0.5% of the patients receiving neoadjuvant therapy), female patients had lower rate of surgical complications and shorter post-operative hospital stays, probably due to differences in host and tumor characteristics such as age, body mass index and preexisting lung disease.^36^ In our cohort, surgical outcomes such as rate of R1 resection and duration of hospital stay across both sexes were largely similar; however, the rate of pneumonectomies was significantly increased in male patients (23% versus 13%, *P* ≤ 0.005). The reason for this higher rate of pneumonectomies is not fully clear as overall stage distribution at baseline between sexes was comparable. However, detailed data on the exact location and especially on the percentage of centrally located tumors could not be retrieved and may have contributed to this result. Importantly, the attrition rate in our cohort was comparable to current phase III studies with neoadjuvant/perioperative chemo-immunotherapy (20%) and did not differ between sexes.

Unfortunately, current neoadjuvant and perioperative NSCLC trials only reported safety and toxicity outcomes for the overall population and not separated by sex. In contrast to previous data suggesting increased toxicity from anticancer therapies in females, rates of ≥grade 3 treatment-related AEs as well as rate of treatment discontinuation due to toxicity did not differ between sexes in our analysis. However, female patients had significantly higher probability of dose modifications due to toxicity (32% versus 23%) compared with males. Several host factors including differences in body composition and drug metabolism result in higher blood levels and greater risk of AEs among females upon body surface-based or body weight-based dosing of chemotherapies or ICIs.^37^

The strengths of our study are the analyses of individual patient data from prospective clinical trials in a patient population exclusively constituted of stage III (with the majority with N2 disease) NSCLC patients. There are some limitations of our trial. The analysis was conducted on data from five different trials, each with varying study designs and treatment strategies. However, the chemotherapy backbone in all five trials was cisplatin and docetaxel, mitigating some of the difficulties of outcome comparisons. Moreover, some treatment strategies considered in past trials may no longer be part of the current standard of care, limiting their applicability to present-day clinical practice.

Although the cisplatin/docetaxel regimens used in our study are no longer the most common combinations, current neoadjuvant treatments for resectable stage III NSCLC are still based on a platinum-based doublet, with cisplatin preferred for fit patients and carboplatin for cisplatin-unfit patients. The primary aim of the chemotherapy is to reduce tumor burden and provide a foundation for the integration of newer agents such as ICI. Thus, while the specific treatment regimens have evolved, the underlying principles remain consistent, and our findings are still likely to be relevant to contemporary clinical practice.

A limitation of our study is the absence of systematic testing for molecular alterations such as *EGFR* or *ALK*, which are now routinely assessed and guide perioperative treatment. As molecular profiling was not standard when these trials were conducted, some patients may have had oncogene-driven tumors which could limit the generalizability of our findings to current practice.

Likewise, despite statistical adjustments for the number of patients with adenocarcinoma, the possibility of a sex-based imbalance in actionable driver mutations (e.g. EGFR, ALK) within our cohort and its impact on key endpoints cannot be excluded. Detailed safety reporting was not available for all trials; however, clinically most relevant data as rate of dose modification and treatment discontinuation due to toxicity could be retrieved for most patients and according to sex. Information on comorbidities and Charlson comorbidity index were not systematically collected, limiting interpretation of the reported increase in non-cancer-related mortality and this should be clearly a focus in future trials.

In conclusion, female patients with resectable stage III NSCLC experience significantly improved survival outcomes such as EFS and OS, without increased treatment-related toxicity. Importantly, the survival benefit was primarily driven by lower non-cancer-related mortality, as cancer-specific mortality was similar between sexes. These results underscore the importance of considering sex-specific factors for the investigation of anticancer treatments in future clinical trials.
