# Supplementary material for: Sex-specific efficacy and safety outcomes in patients with resectable stage III non-small-cell lung cancer (NSCLC) undergoing neoadjuvant therapies: a pooled analysis of the SAKK trials 16/96, 16/00, 16/01, 16/08 and 16/14
Source: ESMO Open. 2025 Nov 10;10(11):105870. doi: 10.1016/j.esmoop.2025.105870 (PMC12651527; doi:10.1016/j.esmoop.2025.105870)
Supplement: Supplementary Material [file mmc1.docx]

Supplementary Tables and Figures

**Figure S1**: Forest plot for multivariable Cox regression for event-free survival for sex, age, smoking status, treatment modality and histology. F; females, M, males; C, chemotherapy; CI, chemo-immunotherapy, CR, chemoradiotherapy, CI, confidence interval


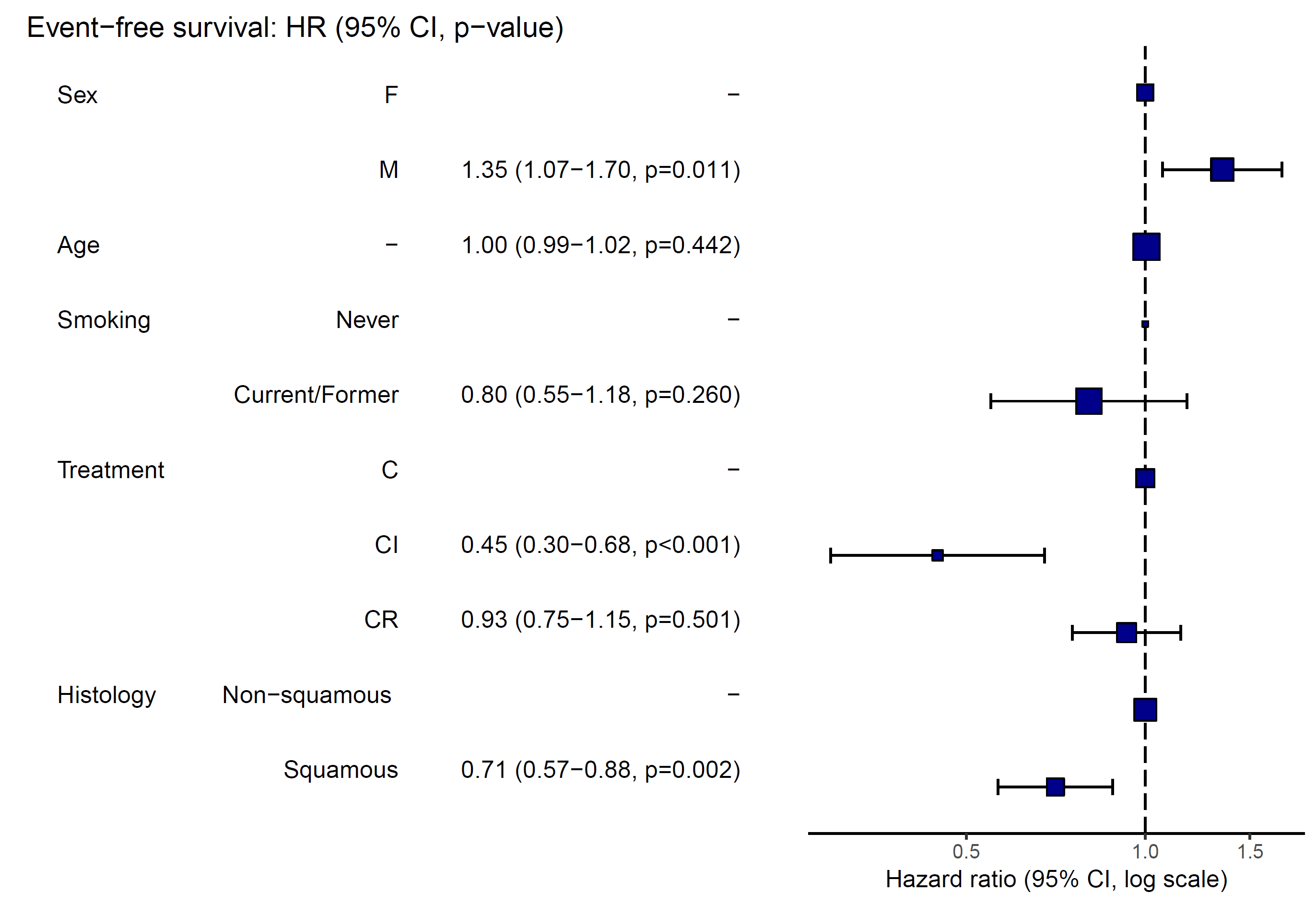


**Figure S2**: Forest plot for multivariable Cox regression for overall survival for sex, age, smoking status, treatment modality and histology. F; females, M, males; C, chemotherapy; CI, chemo-immunotherapy, CR, chemoradiotherapy, CI, confidence interval


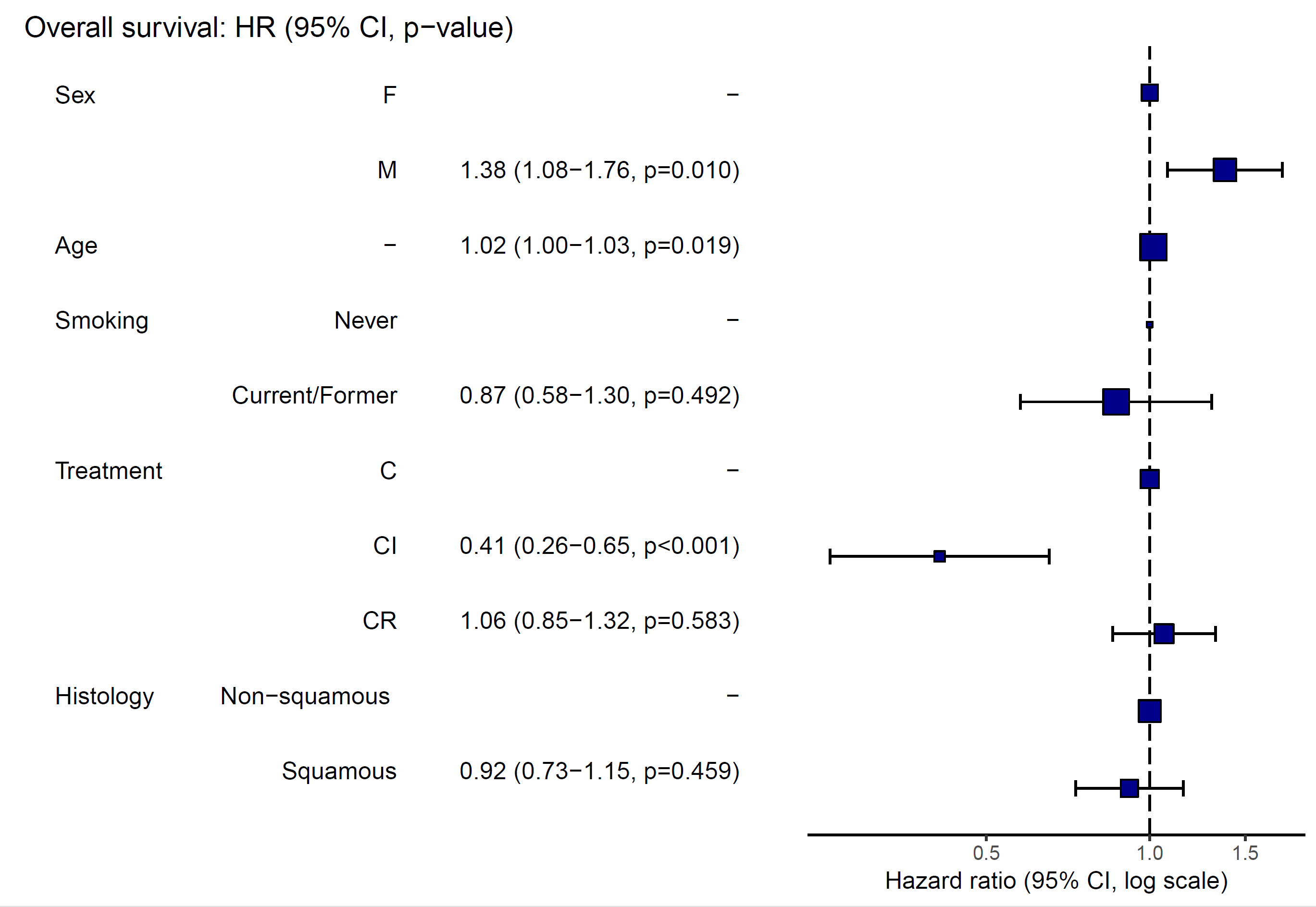


**Table S1:** multivariable Cox regression for event-free survival for sex, age, smoking status, trial participation and histology. F; females, M, males; C, chemotherapy; CI, chemo-immunotherapy, CR, chemoradiotherapy, CI, confidence interval; EFS, event-free survival; OS, overall survival.

| EFS |  | | all | | | HR (univariable) | | HR (multivariable) |
| --- | --- | --- | --- | --- | --- | --- | --- | --- |
| Sex | F | | 158 (31.7) | | | - | | - |
|  | M | | 341 (68.3) | | | 1.32 (1.06-1.64, p=0.015) | | 1.36 (1.08-1.72, p=0.009) |
| Age | Mean (SD) | | 59.6 (8.1) | | | 1.00 (0.99-1.01, p=0.973) | | 1.00 (0.99-1.02, p=0.523) |
| Smoking | Never | | 36 (7.2) | | | - | | - |
|  | Current/Former | | 463 (92.8) | | | 0.76 (0.52-1.10, p=0.140) | | 0.81 (0.56-1.19, p=0.285) |
| Trial | SAKK 16/00 | | 231 (46.3) | | | - | | - |
|  | SAKK 16/01 | | 43 (8.6) | | | 1.05 (0.74-1.50, p=0.774) | | 1.06 (0.74-1.51, p=0.756) |
|  | SAKK 16/08 | | 69 (13.8) | | | 0.97 (0.70-1.35, p=0.867) | | 0.95 (0.69-1.33, p=0.785) |
|  | SAKK 16/14 | | 68 (13.6) | | | 0.52 (0.35-0.76, p=0.001) | | 0.53 (0.36-0.79, p=0.002) |
|  | SAKK 16/96 | | 88 (17.6) | | | 1.11 (0.85-1.45, p=0.434) | | 1.10 (0.84-1.43, p=0.497) |
| Histology | Non-squamous | | 319 (63.9) | | | - | | - |
|  | Squamous | | 180 (36.1) | | | 0.76 (0.62-0.95, p=0.013) | | 0.70 (0.56-0.88, p=0.002) |
| OS | |  | | all | HR (univariable) | | HR (multivariable) | |
| Sex | | F | | 158 (31.7) | - | | - | |
|  | | M | | 341 (68.3) | 1.45 (1.15-1.83, p=0.002) | | 1.36 (1.07-1.74, p=0.013) | |
| Age | | Mean (SD) | | 59.6 (8.1) | 1.01 (1.00-1.03, p=0.067) | | 1.02 (1.00-1.03, p=0.029) | |
| Smoking | | Never | | 36 (7.2) | - | | - | |
|  | | Current/Former | | 463 (92.8) | 0.88 (0.59-1.30, p=0.516) | | 0.88 (0.58-1.32, p=0.524) | |
| Trial | | SAKK 16/00 | | 231 (46.3) | - | | - | |
|  | | SAKK 16/01 | | 43 (8.6) | 1.19 (0.84-1.70, p=0.331) | | 1.19 (0.83-1.69, p=0.350) | |
|  | | SAKK 16/08 | | 69 (13.8) | 1.36 (0.98-1.89, p=0.067) | | 1.30 (0.94-1.81, p=0.116) | |
|  | | SAKK 16/14 | | 68 (13.6) | 0.49 (0.32-0.76, p=0.001) | | 0.49 (0.32-0.75, p=0.001) | |
|  | | SAKK 16/96 | | 88 (17.6) | 1.26 (0.96-1.67, p=0.096) | | 1.20 (0.91-1.59, p=0.202) | |
| Histology | | Non-squamous | | 319 (63.9) | - | | - | |
|  | | Squamous | | 180 (36.1) | 1.04 (0.83-1.29, p=0.752) | | 0.91 (0.72-1.14, p=0.416) | |

**Table S2:** Causes of death other than tumor progression

| **Death cause** | **F (N=19) n (%)** | **M (N=79) n (%)** | **Total (N=98) n (%)** |
| --- | --- | --- | --- |
| Cardioembolic | 3 (16%) | 19 (24%) | 22 (22%) |
|  |  |  |  |
| Infection | 1 (5%) | 7 (9%) | 8 (8%) |
| Postoperative complications | 0 | 3 (4%) | 3 (3%) |
| Respiratory | 1 (5%) | 4 (5%) | 5 (5%) |
| Secondary cancer | 4 (21%) | 7 (9%) | 11 (11%) |
| Sepsis | 0 | 3 (4%) | 3 (3%) |
| Other | 2 (10%) | 12 (15%) | 14 (14%) |
| Missing | 8 (42%) | 24 (30%) | 32 (32%) |

**Table S3:** Patterns of recurrence

| **Patterns of recurrence** | **Overall**  N = 314 | **F**  N = 99 | **M**  N = 215 | **p-value** |
| --- | --- | --- | --- | --- |
| Distant (Distant only and local and distant) | 284 (90%) | 96 (97%) | 188 (87%) | 0.004 |
| Local only | 27 (9%) | 2 (2.0%) | 25 (12%) |  |
| Unknown | 3 (1%) | 1 (1%) | 2 (1%) |  |

**Table S4**: Post-relapse treatment patterns in the SAKK 16/00, 16/08 and 16/14 trials^1^

| **Characteristic** | **Overall**, N = 226 | **F**, N = 74 | **M**, N = 152 | **p-value** |
| --- | --- | --- | --- | --- |
| Systemic further therapy | 120 (53%) | 40 (54%) | 80 (53%) | 0.8 |
| Further radiotherapy | 45 (20%) | 18 (24%) | 27 (18%) | 0.2 |
| 1. Post relapse treatment data were prospectively evaluated in the SAKK 16/08 (69 patients) and SAKK 16/14 (68 patients) trials, and partially in the SAKK 16/00 trial (231 patients). | | | | |

**Table S5**: Surgical outcomes

|  | **F (N=158)** | | | **M (N=341)** | | |  |
| --- | --- | --- | --- | --- | --- | --- | --- |
| **Variable** | **n^1^** | **median** | **(min, max)** | **n^2^** | **median** | **(min, max)** | **p-value^3^** |
| Duration of hospital stay (days) | 85 | 12.0 | (6.0, 76.0) | 194 | 12.0 | (3.0, 134.0) | 0.96 |
| Pneumonectomy |  | 20 | (13%) |  | 77 | (23%) | 0.009 |
| R1 |  | 12 | (8%) |  | 25 | (7%) | 0.92 |
| 30 day mortality |  | 1 | (1%) |  | 3 | (1%) | 0.77 |
| 60 day mortality |  | 2 | (1%) |  | 4 | (1%) | 0.93 |

1. number of female patients (non-missing)

2. number of male patients (non-missing)

3. Pearson’s Chi-square test; Wilcoxon rank-sum test
